# IL-33 signaling is dispensable for the IL-10-induced enhancement of mast cell responses during food allergy

**DOI:** 10.3389/fimmu.2025.1526498

**Published:** 2025-01-28

**Authors:** Dylan Krajewski, Saurav Ranjitkar, Nathan Jordan, Sallie S. Schneider, Clinton B. Mathias

**Affiliations:** ^1^ Department of Pharmaceutical and Administrative Sciences, Western New England University, Springfield, MA, United States; ^2^ Department of Nutritional Sciences, University of Connecticut, Storrs, CT, United States; ^3^ Pioneer Valley Life Sciences Institute, Baystate Medical Center, Springfield, MA, United States

**Keywords:** mast cells, food allergy, IL-10, IL-33, allergy

## Abstract

**Background:**

The IL-33/ST2 axis plays a pivotal role in the development of IgE-mediated mast cell (MC) responses during food allergy. We recently demonstrated that the pleiotropic cytokine, IL-10, not only exerts proinflammatory effects on IgE-mediated MC activation, but also promotes IL-33-induced MC responses. However, whether IL-33 is necessary for IL-10’s proinflammatory effects has not been examined.

**Methods:**

To therefore determine the role of the IL-33/ST2 axis in this pathway, we assessed the effects of IL-10 on IgE-mediated MC activation and food allergy development in wild-type (WT) and ST2^-/-^ mice.

**Results:**

IL-10 stimulation significantly enhanced IL-33 gene expression, ST2 receptor expression, cytokine production, mMCP-1 secretion, and proliferation in IgE and antigen-activated bone marrow-derived MCs (BMMCs) from WT mice. ST2^-/-^ BMMCs exhibited reduced cytokine secretion in response to IgE-dependent activation. However, IL-10 enhanced cytokine production, mMCP-1 secretion, and proliferation in these cells as well. To further assess the role of IL-10, food allergy was induced in WT and ST2^-/-^ mice subjected to antibody-mediated IL-10 depletion. IL-10-depleted WT mice exhibited a significant attenuation in MC-mediated responses to OVA challenge. While ST2^-/-^ mice also exhibited a profound suppression of MC responses, IL-10 depletion had no additional effects. However, ST2^-/-^/IL-10^-/-^ mice exhibited further decreases in OVA-IgE and antigen-specific MC activation compared to ST2^-/-^ mice.

**Conclusion:**

Our data demonstrates that IL-10 can enhance MC responses in both WT and ST2^-/-^ mice, further corroborating its proinflammatory effects on MCs and suggesting that they are not regulated by IL-33 signaling.

## Introduction

Allergic sensitization to food allergens has been steadily increasing in the Western world (1–3). Mast cell (MC) activation induced by food allergen-specific IgE antibodies plays a critical role in the development of the allergic response, leading to various proinflammatory effects including vasodilation, smooth muscle hyperreactivity, and in rare cases, systemic anaphylactic reactions (3–6). Various endogenous and environmental factors are known to exert stimulatory effects on MCs to promote allergic sensitization. Of these, epicutaneous sensitization with food antigens leading to the production of the alarmin cytokine, IL-33, by epithelial cells, has been shown to have a prominent effect on MC activation and function (7, 8). Furthermore, we and others have shown that various Th2-derived cytokines such as IL-3, IL-4, IL-9 and IL-10 can have critical roles in promoting MC activation and function during food allergy (9–16).

We recently demonstrated that the pleiotropic cytokine, IL-10, has unexpected pro-inflammatory effects on MC responses (14, 17, 18). IL-10 promoted the activation and function of both IgE-activated and IL-33-stimulated MCs, leading to enhanced MC responses during food allergy (14, 17), passive anaphylaxis (14), and type 2 inflammation (18). In the absence of IL-10, food allergy development was attenuated, leading to decreased MC expansion and IgE-mediated MC activation (14, 17). Furthermore, the transfer of either IL-10-producing CD4^+^ T cells or WT MCs restored the development of food allergy in IL-10^-/-^ mice (14). The proinflammatory effects of IL-10 on MCs were also observed on bone marrow-derived MCs (BMMCs) in cell culture (14, 18). IL-10 directly promoted the proliferation and survival of these cells and enhanced the effects of IgE and/or IL-33-induced activation leading to increased secretion of granule contents and the production of type 2 cytokines such as IL-13 (14, 18). These effects correlated with enhanced expression of the FcεRI and ST2 receptors on MCs, suggesting that IL-10 enhances the responsiveness of MCs to their respective ligands (14, 18). These data are consistent with several other reports demonstrating similar effects of IL-10 on MCs both during allergic responses and other diseases (19–25).

In a similar vein, we recently also demonstrated a critical role for IL-33 in inducing MC expansion and activation during food allergy development (26). IL-33 is a potent stimulator of MCs and can promote both IgE-dependent and independent MC-mediated inflammation (27–32). MCs constitutively express the IL-33 receptor, ST2 (also called ST2L), which promotes MC differentiation and survival, induces antigen-independent degranulation, and elicits the production of cytokines such as IL-6 and IL-13 (27, 33–35). Furthermore, MCs activated with IgE and antigen can produce IL-33 (35, 36) whereas IL-33-responding MCs have been shown to potentiate IgE-mediated responses (7, 8, 36–42).

We therefore wondered whether the effects of IL-10 on MCs may be regulated by IL-33 signaling and investigated the role of IL-10 on MC activation and function during food allergy development in ST2^-/-^ mice. Our data demonstrate that IL-10 enhances MC responses in both WT and ST2^-/-^ mice, suggesting that IL-33 signaling is not required for IL-10’s effects either during IgE-mediated MC activation or food allergy. Furthermore, while both IL-33 and IL-10 were independently required for full MC responsiveness during food allergy, IL-10 deficiency further decreased MC responses in ST2^-/-^ mice. Collectively, these data demonstrate that IL-10 is critical for allergen-specific MC responses in mice and that its proinflammatory effects extend beyond the IL-33/ST2 signaling axis.

## Methods

### Animals

BALB/c mice were purchased from The Jackson Laboratory and Envigo and used as WT controls in all experiments. IL-10^-/-^ mice on the BALB/c background were purchased from The Jackson Laboratory. ST2^-/-^ mice on the BALB/c background are a kind gift of Dr. Andrew McKenzie, Medical Research Council, United Kingdom and Drs. Paul Bryce and Gurjit Khurana Hershey. IL-10^-/-^ and ST2^-/-^ mice were crossed to generate ST2^-/-^/IL-10^-/-^ mice. All mice were bred in our facilities and all animal research was performed as approved by the IACUCs at the respective institutions, Western New England University (protocol no. 2019-S1) and the University of Connecticut (protocol no. A22-048).

### BMMC culture

BMMCs were generated from naïve WT BALB/c and ST2^-/-^ animals as previously described (14). Briefly, bone marrow cells were collected from the tibia and femurs of animals and cultured with 10 ng/ml of rIL-3 and rSCF (Shenandoah) for >4 weeks. Harvested BMMCs were positive for c-Kit and FcϵRI.

### BMMC activation

1 million BMMCs/ml were cultured in triplicate with 10 ng/ml IL-3 and SCF. Cells were activated by pre-sensitizing with 1 µg/ml DNP-IgE (clone SPE7, Sigma) or vehicle (medium), followed by treatment with 200 ng/ml DNP-BSA (14, 43). Some groups of cells were treated with 20 ng/ml of rIL-10 and/or rIL-33 (Biolegend) for various time periods (including 6 hours and 24hrs) prior to challenge with DNP-BSA. Thirty minutes to an hour after activation with DNP-BSA, cells were collected for isolation of RNA and cDNA was created. The cDNA was then used to assess the expression of various cytokine genes as described in the manuscript. In other experiments, supernatants were collected 6-24h later for the assessment of secreted cytokines by ELISA.

### Quantitative PCR analysis and ELISAs

Quantitative RT-PCR was performed as previously described using Taqman probes (14, 43). The expression of cytokine genes (IL-4, IL-5, IL-13, IL-10, IL-33, IFN-γ) was calculated relative to GAPDH transcripts. ELISAs for mMCP-1 (Thermofisher), IL-4, IL-5, IL-6, TNF-α and IFN-γ (Biolegend), IL-13 (R&D Systems), and OVA-IgE were performed according to manufacturers’ protocols as previously described (14, 43).

### BMMC proliferation

BMMCs were grown in rIL-3 and rSCF as described above. Some groups of cells were treated with 20 ng/ml of rIL-10. Cells were counted daily for 1-3 days and live cells were enumerated on the basis of trypan blue exclusion or using a quantitative tetrazolium reduction cell proliferation assay (MTS assay kit by Abcam). Cell proliferation was calculated based on OD using the formula: (OD of samples – OD of untreated control)/(OD of untreated control × 100).

### β-hexosaminidase assay

BMMCs were cultured in the presence or absence of 20 ng/ml rIL-10 for 24 hours. Cells were activated with IgE and antigen and β-hex activity was assessed as previously described (44).

### Flow cytometry

Cultured BMMCs were resuspended in staining medium (SM) containing 1X HBSS, HEPES buffer, and 2% fetal calf serum and incubated with mAbs against mouse c-Kit, FcεRI, and ST2 (Biolegend). Stained cells were then washed and assessed phenotypically by flow cytometry as previously described (18).

### Food allergy regimen

To induce food allergy, WT, IL-10^-/-^, ST2^-/-^ and IL-10/ST2^-/-^ mice were *i.p.* immunized with 50 μg chicken egg OVA in 1 mg alum twice (two weeks apart), as previously described (14, 43, 45). Four weeks later, mice were challenged *i.g.* with 50 mg OVA on 6 alternating days. Control animals were *i.p.* sensitized but not challenged with OVA. To supplement knockout data, some groups of mice were also treated with blocking antibodies for IL-10. In these experiments, mice were treated *i.p.* with 100 μg purified anti-IL-10 (Biolegend) 6 different times immediately prior to OVA challenges. Mice were sacrificed one hour after the 6^th^ challenge with OVA, and food allergy parameters were assessed as previously described (14, 43, 46). Blood was collected for evaluation of antibodies and mMCP-1 in serum. Jejunum was collected for histological assessment of MCs and evaluation of cytokine gene expression by RT-PCR as described above.

### Measurement of intestinal anaphylaxis

Intestinal anaphylaxis was assessed in challenged mice by scoring the percentage of animals exhibiting allergic diarrhea for one hour after OVA challenge (14, 46).

### Histological analysis and enumeration of MCs

Intestinal MCs were enumerated as we have previously described (14). Briefly, paraffin-embedded jejunal sections were stained with chloroacetate esterase (CAE) and MCs were counted in complete cross-sections. Data are represented as the average numbers of MCs in 3 high-powered fields (HPF).

### Passive anaphylaxis

WT and ST2^-/-^ were sensitized *i.v.* with 6 μg DNP-IgE (clone SPE7, Sigma). 24h later, they were challenged *i.v*. with 75 μg of DNP-BSA, and changes in core body temperature were recorded using subcutaneously placed transponders (Biomedic Data Systems). To assess the effects of anti-IL-10 on the development of passive anaphylaxis, some mice were injected *i.p.* with 400 μg rIL-10 concurrently with the DNP-IgE injection.

### Statistical analysis

Data are expressed as mean plus or minus standard error of mean, unless stated otherwise. Statistical significance comparing two groups of mice was determined using the unpaired or paired Student’s t-test as appropriate. Two-way analysis of variance was used to calculate differences between multiple groups.

## Results

### IL-10 enhances ST2 expression and promotes cytokine production in IL-33-stimulated MCs

**Figure 1:**
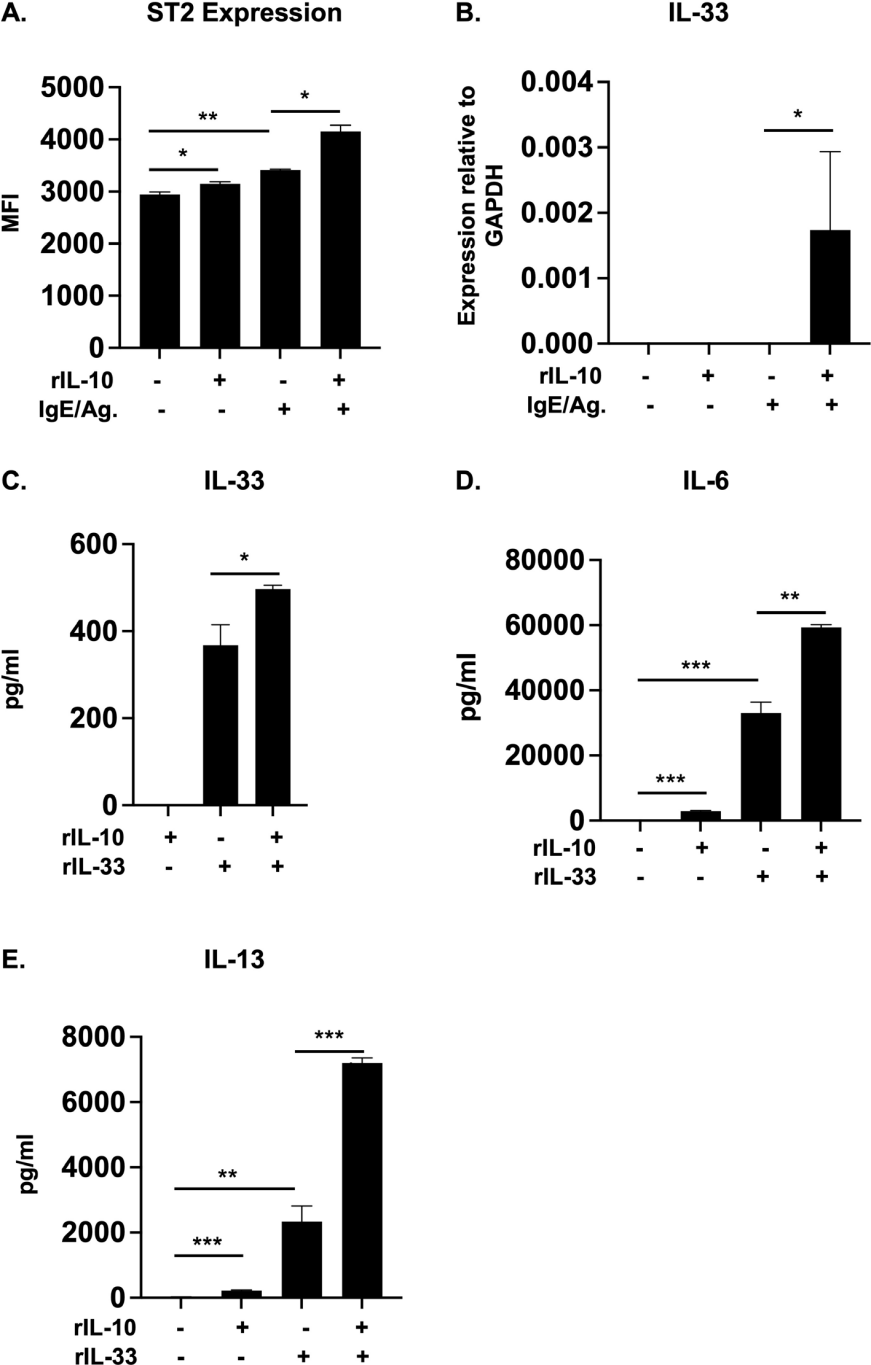
IL-10 enhances ST2 and IL-33 expression and promotes IL-33-mediated cytokine production in BMMCs. **(A, B)** WT BMMCs were cultured with rIL-10 for 24h and activated with IgE/Ag for 1h. **(A)** The expression of ST2 on treated cells was evaluated using flow cytometry. Median fluorescence intensity (MFI) is shown. **(B)** IL-33 mRNA levels relative to GAPDH are shown. **(C-E)** WT BMMCs were cultured with either rIL-10, rIL-33, or both for 7 days. **(C)** IL-33 levels in supernatant are shown. **(D, E)** IL-6 and IL-13 levels after 7 days. Data are representative of >3 experiments. *p<0.05; **p<0.001; ***p<0.0001 (*t*-test).

We have previously demonstrated that IL-10 can enhance FcεRI expression and promote IgE-mediated activation in MCs (14). Similarly, we recently observed that IL-10 can also significantly enhance the expression of the IL-33 receptor, ST2, and promote IL-33-induced type 2 cytokine production (18). As shown in **Figure 1A**, treatment with IL-10 enhanced ST2 expression on WT BMMCs (**Figure 1A**). This was further increased in cells that were cultured with IL-10 and activated with IgE/Ag, suggesting that IL-10 can enhance IL-33 responsiveness during IgE-mediated activation (**Figure 1A**).

IL-33 signaling also plays a critical role in the development of allergic responses and endogenously produced IL-33 has been shown to regulate IgE-mediated MC activation (47, 48). To therefore further evaluate the effects of IL-10, we assessed whether IL-10 can also enhance IL-33 production in IgE/Ag-activated cells. As observed in **Figure 1B** (and data not shown), while we could not detect any IL-33 protein secretion, IL-10 significantly enhanced the transcriptional levels of IL-33 in IgE-activated cells. We have previously shown that IL-10 can also enhance the production of cytokines such as IL-6 and IL-13 in IL-33-stimulated MCs (18). We therefore wondered whether IL-10 may have similar effects on IL-33 production in these cells. Interestingly, as observed in **Figure 1C**, IL-10 pre-treatment significantly enhanced IL-33 protein secretion in IL-33-treated BMMCs. Similarly, increased secretion of the cytokines IL-6 and IL-13 (**Figures 1D, E**) was also observed, suggesting that crosstalk between IL-10 and IL-33 may serve to further potentiate MC responses.

### IL-10 enhances the IgE-mediated activation of ST2^-/-^ BMMCs

**Figure 2:**
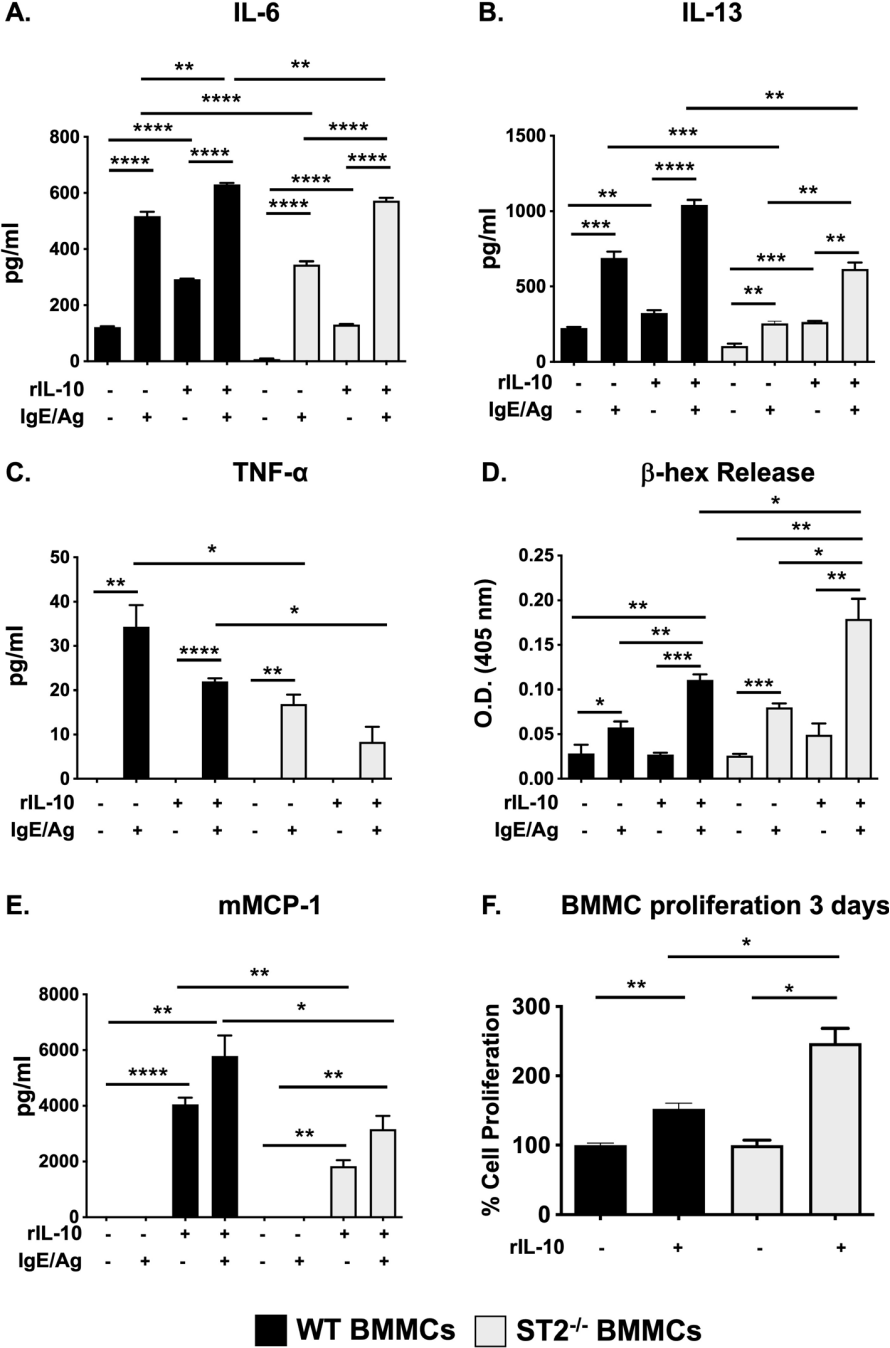
IL-10 promotes the IgE-mediated activation of ST2^-/-^ BMMCs. **(A-E)** WT and ST2^-/-^ BMMCs were cultured with or without rIL-10 for 24h and subsequently activated with IgE and antigen. **(A-C)** Supernatants were collected and cytokine secretion was assessed. **(D)** β-hex activity was measured in supernatants. **(E)** mMCP-1 release was assessed. **(F)** BMMCs were cultured with rIL-10 for 3 days and an MTS assay was performed to assess proliferation. Percent cell proliferation is shown. Data are representative of 2 experiments. *p<0.05; **p<0.01; ***p<0.001; ****p<0.0001 (*t*-test).

To further investigate whether IL-33 signaling is necessary for IL-10’s effects, we next assessed cytokine production in unactivated and IgE-activated ST2^-/-^ BMMCs. As previously observed (14, 18) and shown in **Figures 2A-C**, IL-10 significantly enhanced the production of IL-6 and IL-13 in both unactivated and IgE-activated WT BMMCs. Similarly, IL-10 pre-treatment also enhanced IL-6 and IL-13 production in unactivated ST2^-/-^ BMMCs, suggesting that IL-33 signaling is not required for its effects. In general, ST2^-/-^ BMMCs exhibited reduced cytokine responses after IgE-mediated activation (**Figures 2A-C**), although some variability was observed depending on experimental conditions (**Supplementary Figures S1A-C**). Pre-treatment with IL-10 enhanced the production of both IL-6 and IL-13 but not TNF-α in IgE-activated ST2^-/-^ cells. A similar pattern was also observed after long-term culture (three days) with rIL-10 as we have previously shown (**Supplementary Figures S1A-C**). Taken together, these data suggest that IL-10 may further modulate the function of MCs *in vivo*, and that its effects on IgE-activated MCs are independent of IL-33 signaling. Next, we also assessed the effects of IL-10 on MC degranulation and murine MC protease (mMCP)-1 secretion. As observed in **Figure 2D**, both WT and ST2^-/-^ BMMCs exhibited comparable levels of β-hex activity in response to IgE activation. IL-10 pre-treatment enhanced β-hex release in both cell types and this was higher in ST2^-/-^ BMMCs compared to their WT counterparts. This suggests that while IL-10 can promote MC degranulation independently of IL-33 signaling, endogenous IL-33 may regulate some of its effects. Similarly, as previously shown by us (18), IL-10 also enhanced mMCP-1 secretion in both WT and ST2^-/-^ resting BMMCs (**Figure 2E**). However, lower mMCP-1 levels were observed in IL-10-treated ST2^-/-^ BMMCs compared to WT cells. Interestingly, no mMCP-1 secretion was observed in either WT or ST2^-/-^ BMMCs after activation with IgE and antigen (**Figure 2E**). Instead, IL-10 stimulation led to mMCP-1 secretion in these cells as well (**Figure 2E**). Finally, we have previously shown that IL-10 can also promote MC proliferation during cell culture (14, 18). To assess whether IL-33 signaling may be required in this process, we next also assessed the proliferation of both WT and ST2^-/-^ BMMCs. As observed in **Figure 2F**, IL-10 enhanced the proliferation of BMMCs from both strains. Furthermore, greater proliferation was observed in ST2^-/-^ BMMCs treated with IL-10. While the mechanism for this is unclear, these data further underscore the role of IL-10 and suggest that IL-33 signaling is not required for its effects.

### Allergic responses to enteral ovalbumin administration are suppressed in ST2^-/-^ mice

Recently, IL-33 has emerged as a critical mediator of MC responses during food allergy (8, 26, 49–51). To further investigate the crosstalk between IL-10 and IL-33, and whether IL-10’s effects on IgE-induced MC responses may depend on IL-33, we assessed the development of food allergy in WT and IL-10-depleted ST2^-/-^ mice using an ovalbumin (OVA)-induced model of intestinal anaphylaxis. We and others have previously shown that the development of food allergy in this model is IgE and MC-dependent (9, 14, 43, 46, 52). Similarly, we recently also demonstrated a critical role for IL-33 in inducing MC responses in this model (26). Lastly, using both IL-10^-/-^ mice (14) as well as pharmacological blockade of IL-10 (17), we have shown that MC responses and the development of intestinal anaphylaxis in this model are also IL-10-dependent. We therefore hypothesized that this would be a good system to assess the roles of IL-10 and IL-33 and their interrelated effects on MCs.

**Figure 3:**
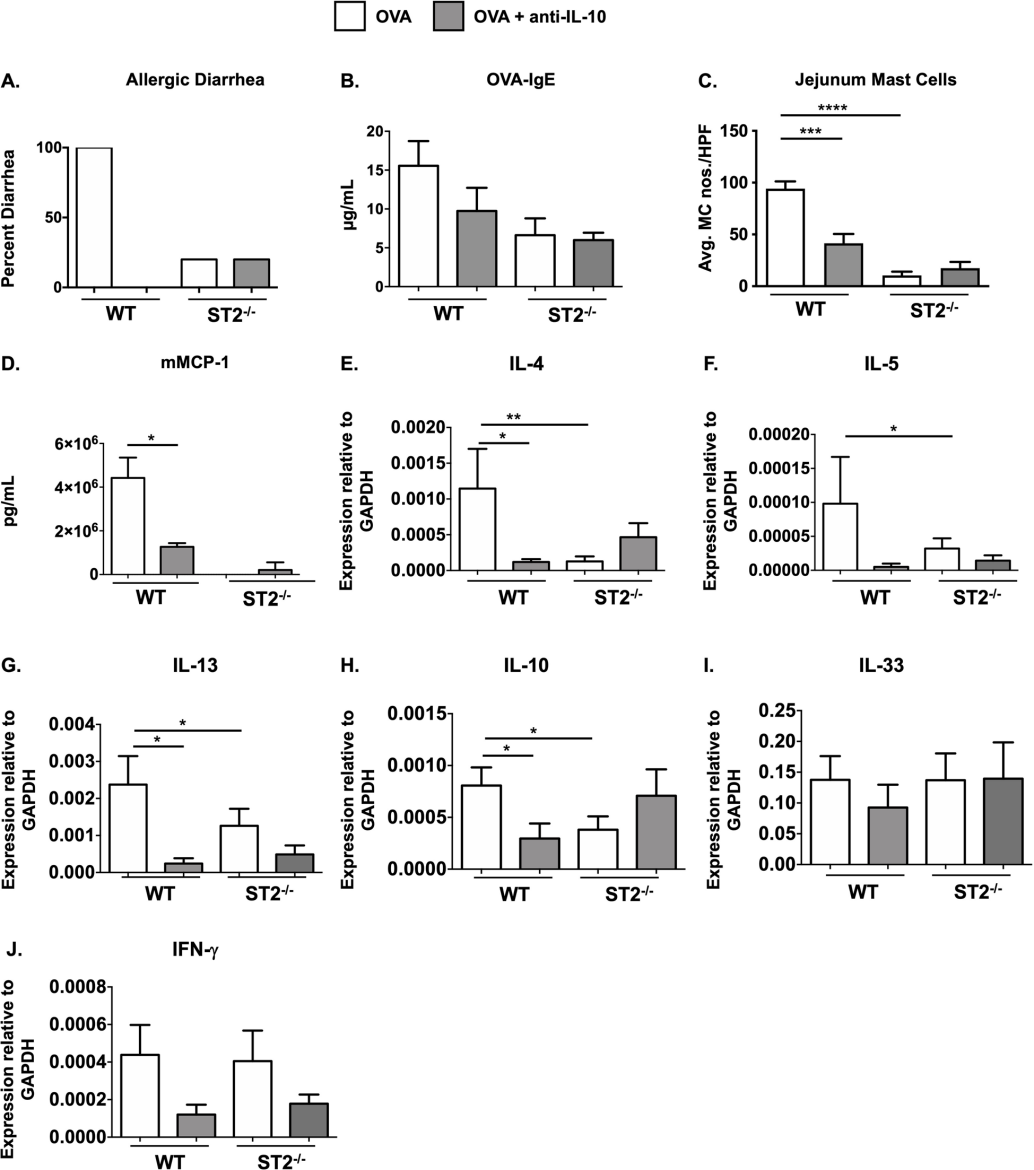
ST2^-/-^ mice exhibit reduced MC responses during food allergy and IL-10 depletion has no further effects. WT BALB/c and ST2^-/-^ mice were sensitized and challenged with OVA as described in Methods. Some groups of animals were treated with anti-IL-10 daily during the challenge phase. One hour after the 6^th^ OVA challenge, mice were sacrificed and the following parameters were measured: **(A)** occurrence of diarrhea; **(B)** serum OVA-IgE levels; **(C)** CAE^+^ MCs in the jejunum; **(D)** serum mMCP-1 levels; **(E-J)** jejunal mRNA expression. Data are representative of 2 experiments. n=5-7 mice/group. *p<0.05; **p<0.01; ***p<0.001; ****p<0.0001 (*t*-test).

Briefly, WT and ST2^-/-^ mice were *i.p.* sensitized with chicken egg OVA and alum as previously described and subsequently challenged orally with OVA to induce the development of intestinal anaphylaxis. As demonstrated in **Figure 3A** and **Supplementary Figure S2A**, WT BALB/c mice developed profuse diarrhea after the sixth oral gavage, accompanied by a robust OVA-specific IgE-mediated response (**Figure 3B**, **Supplementary Figure S2B**). Histological analysis using chloroacetate esterase staining revealed a significant recruitment of mature degranulating MCs to the small intestine of WT OVA mice compared to unchallenged controls (**Figure 3C**, **Supplementary Figure S2C**). Furthermore, assessment of MC activation in WT mice revealed the presence of elevated levels of serum mMCP-1, a marker correlated with the degranulation of mucosal MCs (**Figure 3D**, **Supplementary Figure S2D**). In contrast to these positive markers of food allergy in WT mice and as previously observed by us (26), ST2^-/-^ animals did not develop allergic diarrhea or exhibit MC-mediated activation, suggesting that the IgE and MC-dependent effects of food allergy require IL-33 signaling (**Figures 3A-D**).

### Pharmacological IL-10 depletion has no additional effects on MC responses in ST2^-/-^ mice

To next examine the effects of IL-10 depletion, some groups of mice were treated with anti-IL-10 during the acute, MC-dependent, challenge phase of the model as described above. As expected and consistent with our previous findings (17), anti-IL-10 treatment in WT mice significantly attenuated MC responses including decreased allergic diarrhea (**Figure 3A**), OVA-specific IgE production (**Figure 3B**), intestinal MC numbers (**Figure 3C**) and mMCP-1 levels (**Figure 3D**). Interestingly, however, treatment with anti-IL-10 had no additional effects in ST2^-/-^ mice, including changes in allergic diarrhea, OVA-IgE or MC numbers, or intestinal cytokine expression in ST2^-/-^ mice (**Figures 3A-J**). mMCP-1 levels were below the limits of detection in ST2^-/-^ animals, except in the case of one mouse (**Figure 3D**). These data suggest that IL-33 signaling is not required for IL-10’s effects on MCs in the food allergy model.

To next assess the effects of IL-10 on intestinal type 2 cytokine expression, we examined jejunal tissue from experimental animals for various cytokine transcripts. As we have previously reported, while the expression of IL-4, IL-5, IL-13, and IL-10 was increased in the jejunae of allergic WT mice, the induction of these cytokines was significantly decreased in both ST2^-/-^ and anti-IL-10-treated WT mice (**Figures 3E-H**). In contrast, no significant differences were observed between control and anti-IL-10-treated ST2^-/-^ mice. Furthermore, no significant differences were observed in the expression of IFN-γ or IL-33 between any of the groups (**Figures 3I, J**).

We have previously shown that ST2^-/-^ mice exhibit reduced IL-4 responses during food allergy which may affect the development of antigen-specific IgE (26). Similarly, IL-10 has also been shown to play an important role in the development of IgE production (53–55). To therefore ascertain whether the effects of anti-IL-10 in our system may be related to reduced IgE responses as opposed to functional defects in MCs, we next assessed the development of IgE-mediated passive anaphylaxis in anti-IL-10-treated naïve WT and ST2^-/-^ mice. As observed in **Supplementary Figure S3**, IgE-sensitized WT mice exhibited significant drops in core body temperature after intravenous antigen administration. In contrast, the induction of hypothermia was delayed in ST2^-/-^ mice. Anti-IL-10 treatment attenuated the development of passive anaphylaxis in WT mice, suggesting that basal IL-10 levels may prime MC responsiveness to IgE-mediated activation. This is also consistent with our previous observation demonstrating that exogenous IL-10 priming can enhance IgE-mediated passive anaphylaxis (14). However, similar effects were not observed in ST2^-/-^ mice. While anti-IL-10 treatment initially had no effect in these mice, a more sustained hypothermic response was observed in anti-IL-10-treated ST2^-/-^ mice, suggesting that other factors may also be involved in these animals, including effects on basophils and other cell types. Taken together, these data suggest that the reduced MC responses in both ST2^-/-^ mice and anti-IL-10-treated WT mice during food allergy occur irrespectively of IgE levels and may result directly as a consequence of functional deficiencies in MCs.

### MC responses and food allergy are further diminished in ST2^-/-^ mice deficient in IL-10

**Figure 4:**
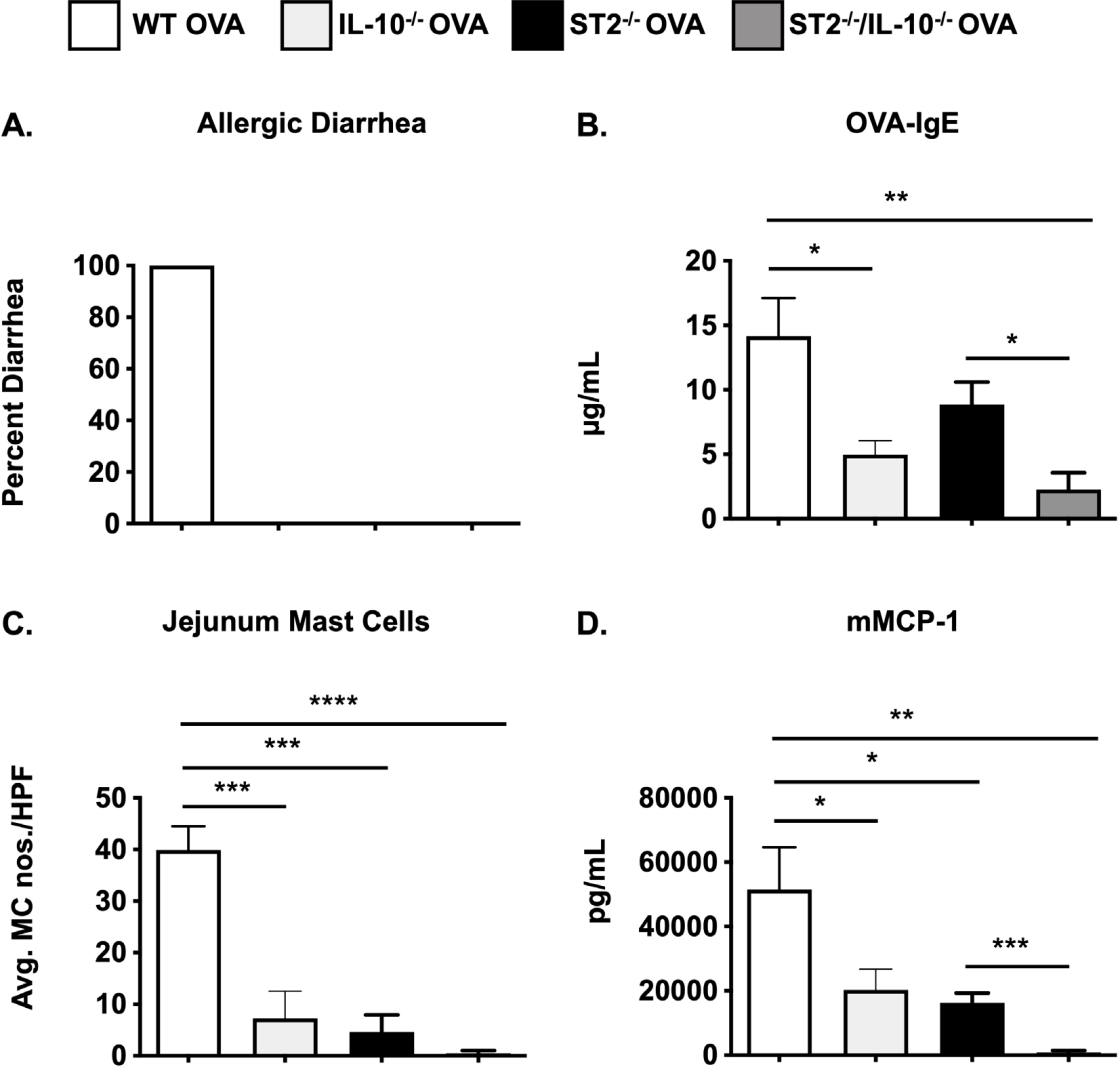
MC responses are further decreased in ST2/IL-10^-/-^ mice. WT BALB/c, IL-10^-/-^, ST2^-/-^, and ST2/IL-10^-/-^ mice were sensitized and challenged with OVA as described in Methods. One hour after the 6^th^ OVA challenge, mice were sacrificed and the following parameters were measured: **(A)** occurrence of diarrhea; **(B)** serum OVA-IgE levels; **(C)** CAE^+^ MCs in the jejunum; **(D)** serum mMCP-1 levels. n=5-7 mice/group. *p<0.05; ***p<0.001; ****p<0.0001 (*t*-test).

Considering that ST2^-/-^ mice exhibit profoundly attenuated MC responses during food allergy and to rule out any variability that may be associated with the pharmacological depletion of IL-10, we next generated mice with a genetic deficiency in both IL-10 and ST2, and examined their susceptibility to the development of food allergy. As observed in **Figure 4**, neither ST2^-/-^ nor ST2^-/-^/IL-10^-/-^ OVA-sensitized mice developed diarrhea in response to OVA challenges (**Figure 4A**). As expected, OVA-IgE levels (**Figure 4B**) and MC responses including intestinal MC numbers and mMCP-1 levels were decreased in the absence of IL-33 signaling (**Figures 4C, D**). To our surprise, however, genetic deletion of IL-10 in these animals further decreased the levels of OVA-IgE and mMCP-1 levels (**Figures 4B, D**). This was also accompanied by a decrease in the number of intestinal MCs (**Figure 4C**). These data suggest that mice with an intrinsic deficiency in both ST2 and IL-10 are further protected from food allergy development and that IL-10 can regulate the IgE-mediated activation of MCs even in the absence of IL-33 signaling.

## Discussion

In this study, we sought to investigate whether the proinflammatory effects of IL-10 on IgE-mediated MC activation may be regulated by IL-33. While we found that IL-10 can promote IL-33 gene expression and its secretion in BMMCs, it could enhance IgE-mediated activation even in ST2^-/-^ BMMCs, demonstrating that IL-33 signaling is not required for IL-10’s effects. These observations were extended *in vivo*, where we found that although the IL-33/ST2 axis was required for MC responses during food allergy, IL-10’s proinflammatory effects on MCs were independent of IL-33 signaling. Instead, IL-10 also promoted IgE-dependent responses in ST2^-/-^ mice. These data further confirm the role of IL-10 as a potent costimulator of MC responses that can prime MCs for IL-33 responsiveness, but the effects of which are not dependent on endogenous IL-33 signaling.

IL-10 is a pleiotropic cytokine that is known to have both pro- and anti-inflammatory effects (56–58). We and others have demonstrated that IL-10 can play critical roles during allergic responses and exert pro-inflammatory effects on MCs and other cells. IL-10 was initially identified as a MC stimulator and shown to promote mMCP-1 expression in MCs (19, 20, 59–62). Similarly, IL-10 can also induce IL-9 production by IgE-cross-linked MCs (21). While some studies had suggested that IL-10 may induce suppression of IgE-mediated signaling in MCs (63, 64), more recently, several investigators including us, have shown that IL-10 can promote MC responses during food allergy (14), enhance STAT3 and miR-155-induced IgE-mediated activation (23), and promote MC expansion and activity during small bowel cancer (24). Several studies also suggest that IL-10 can have mixed (pro- and anti-inflammatory) effects during allergic inflammation. Interestingly, some of these demonstrated that IL-10 can promote the development of airway hyperresponsiveness, mucus metaplasia, IL-5 production, eosinophilia, dendritic cell polarization and a Th2-skewed phenotype in allergic mice (53, 54, 65–73). More recently, IL-10 was shown to both be required for the development of allergen-specific T_H_2 cells as well as promote effector T cell function (74). Similarly, B cell-derived IL-10 promoted allergic sensitization during asthma (75).

In a recent report, we demonstrated that IL-10 can not only promote IgE-dependent MC responses, but also potently co-stimulate IL-33-stimulated MCs by increasing ST2 responsiveness (18). These observations suggest that IL-10’s proinflammatory effects on MCs may be global in nature and not restricted to IgE-allergen crosslinking. Considering that MCs have previously been shown to produce IL-33, we therefore wondered whether MC-derived IL-33 may regulate IL-10’s effects. Interestingly, while in our hands, we could not detect IL-33 protein secretion in IgE-activated cells, IL-10 pre-treatment enhanced IL-33 mRNA expression in these cells. Similarly, IL-10 treatment also enhanced the production of IL-33 in IL-33-treated BMMCs. Finally, while ST2^-/-^ MCs exhibited reduced cytokine responses when activated with IgE and antigen, IL-10 was able to enhance cytokine secretion, degranulation, mMCP-1 release, and proliferation in these cells as well. Collectively, these data suggest that endogenous IL-33 signaling in MCs is not required for IL-10’s proinflammatory effects on MC activation or function.

These observations prompted us to further explore the interactions of IL-10 in the context of IgE and IL-33-mediated signaling during physiological allergic responses *in vivo*. The role of IL-33 in regulating MC responses during allergic inflammation is well-established (30, 33, 48, 76). IL-33-responding MCs have been shown to promote IgE-dependent responses, enhance proinflammatory cytokine production, and facilitate allergic symptoms including bronchoconstriction, IL-13-induced mucus secretion, and systemic anaphylaxis (7, 8, 36–42). Conversely, MCs and their mediators also amplify IL-33-mediated inflammation, by enhancing the recruitment of leukocytes and promoting the activation of group 2 innate lymphoid cells (ILC2s) (77). Furthermore, release of IL-33 by epithelial cells early during allergic inflammation results in the activation of both MCs and ILC2s, leading to either the enhancement of inflammation or its regulation depending on the context and the type of allergen (7, 41, 78, 79).

Several studies have demonstrated the importance of IL-33 signaling during food allergy. However, depending on the model systems used, divergent effects on MC activation, intestinal MC numbers, and allergen-specific Th2 cells have been observed. Food allergy induction in epicutaneously sensitized mice or mice with alterations in IL-4 signaling was attenuated in ST2^-/-^ mice, suggesting that IL-33-mediated signals are critical for the development of oral anaphylaxis in mice, and that IL-33 promotes food anaphylaxis by targeting MCs or ILC2s (7, 8, 49–51, 80). However, in the absence of ST2, while cutaneously sensitized mice were protected from anaphylaxis due to decreased MC activation and mMCP-1 levels, no changes in systemic and intestinal MC numbers were observed, suggesting normal MC expansion (8). Similarly, no differences in T_H_2 cytokines or IgE antibodies were also observed (8). In contrast, other investigators observed decreased T_H_2 responses and reduced MC accumulation during allergic responses (including asthma) in ST2^-/-^ mice (31, 50, 51, 81). In our hands, we observed both decreased MC activation and expansion as well as Th2 cytokine gene expression in the intestinal anaphylaxis model (26). While these dichotomous observations may be a consequence of different experimental models and sensitization regimens, they further strengthen the importance of IL-33 in modulating MC responses *in vivo*.

Our findings also further confirm the importance of IL-10 in regulating MC responses during the development of food allergy. As we have previously observed, both IL-10-deficiency and anti-IL-10 treatments attenuated allergic symptoms, accompanied by decreased MC activation, T_H_2 cytokine expression, and intestinal MC expansion (14, 17). In this study, we demonstrate that IL-10 is able to regulate IgE-mediated MC proliferation and activation as well as MC responses during food allergy even in the absence of IL-33 signaling, suggesting that IL-33 is dispensable for IL-10’s effects. Interestingly, while this was suggested by both antibody-mediated depletion as well as genetic deletion of IL-10, a few subtle differences were observed between the two strategies. Anti-IL-10 treatment in ST2^-/-^ mice had no further effects on IgE levels, intestinal MC numbers, MC activation and jejunal T_H_2 cytokine expression (**Figure 3**). In contrast, genetic deletion of IL-10 led to a greater reduction in MC responses in ST2^-/-^ mice (**Figure 4**). While these differences may be due to the variability involved with antibody-mediated targeting approaches, they may also point to different effects of IL-10 during the allergen sensitization and challenge phases as the anti-IL-10 treatments were only performed during OVA challenge. In this context, while IL-10 is known to promote the induction of antigen-specific IgE (53) and IL-33 is a potent stimulator of Th2 cells (82), the passive anaphylaxis study in **Supplementary Figure S3**, suggests that their individual effects on MCs are independent of circulating IgE levels. Taken together, the data from both model systems collectively suggests that IL-10’s effects on MCs can extend beyond IL-33 signaling. Furthermore, while both IL-33 and IL-10 may act as independent variables that regulate MC function during food allergy, these data also point to a potential for synergistic control if it were necessary.

In summary, our study not only further corroborates the proinflammatory role of IL-10 on MCs during allergic sensitization but suggests that it has unilateral effects on MCs that are independent of other MC stimulators such as IL-33. The mechanisms by which IL-10 exerts these effects need to be further investigated.

## Data Availability

The original contributions presented in the study are included in the article/**Supplementary Material**. Further inquiries can be directed to the corresponding author.

## Supplementary material

Supplementary Figure 1 Long-term IL-10 culture enhances cytokine secretion in IgE-activated ST2^-/-^ BMMCs. **(A-C)** WT and ST2^-/-^ BMMCs were cultured with or without rIL-10 for 3 days and subsequently activated with IgE and antigen. Supernatants were collected and cytokine secretion was assessed. *p<0.05; **p<0.01; ***p<0.001 (*t*-test).

Supplementary Figure 2 MC responses and food allergy development are attenuated in ST2^-/-^ mice. BALB/c and ST2^-/-^ mice were sensitized and challenged with OVA as described in Methods. One hour after the 6^th^ challenge, the following parameters were measured: **(A)** occurrence of diarrhea; **(B)** serum OVA-IgE levels; **(C)** CAE^+^ MCs in the jejunum (magnification: 40X; arrows depict MCs); **(D)** serum mMCP-1 levels. Data are representative of >3 experiments. n=5-7 mice/group. ND, not detected. *p<0.05; **p<0.01 (*t*-test).

Supplementary Figure 3 Anti-IL-10 treatment attenuates IgE-mediated passive anaphylaxis in WT mice. Naïve BALB/c and ST2^-/-^ mice were injected i.v. with 6 μg DNP-IgE. Some groups of mice were treated i.p. with 400 μg anti-IL-10. 24h later, all mice were injected i.v. with 75 μg DNP-BSA and changes in body temperature were measured. n=4-5 mice/group. **p<0.05 for the WT group compared with WT plus anti-IL-10-treated mice by 2-way ANOVA; ***p<0.05 for the WT group compared with ST2^-/-^ mice and the ST2^-/-^ group compared with anti-IL-10-treated ST2^-/-^ mice by 2-way ANOVA.
