# Supplementary figures and images for: IL-33 signaling is dispensable for the IL-10-induced enhancement of mast cell responses during food allergy
Source: Front Immunol. 2025 Jan 28;16:1526498. doi: 10.3389/fimmu.2025.1526498 (PMC11810977; doi:10.3389/fimmu.2025.1526498)

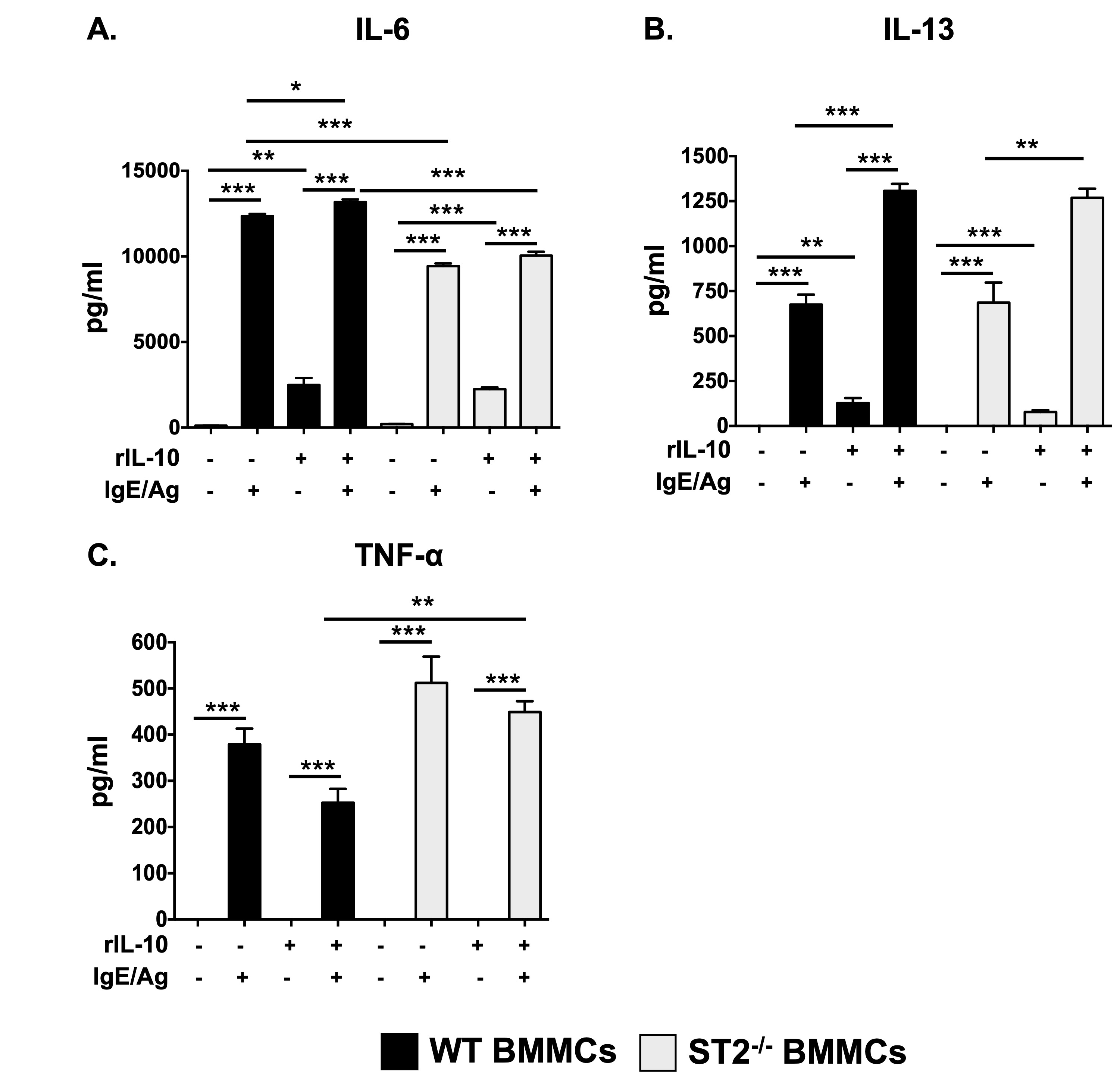

Supplement: Supplementary Figure 1 — Long-term IL-10 culture enhances cytokine secretion in IgE-activated ST2-/- BMMCs. (A-C) WT and ST2-/- BMMCs were cultured with or without rIL-10 for 3 days and subsequently activated with IgE and antigen. Supernatants were collected and cytokine secretion was assessed. *p<0.05; **p<0.01; ***p<0.001 (t-test). [file Image1.jpeg]

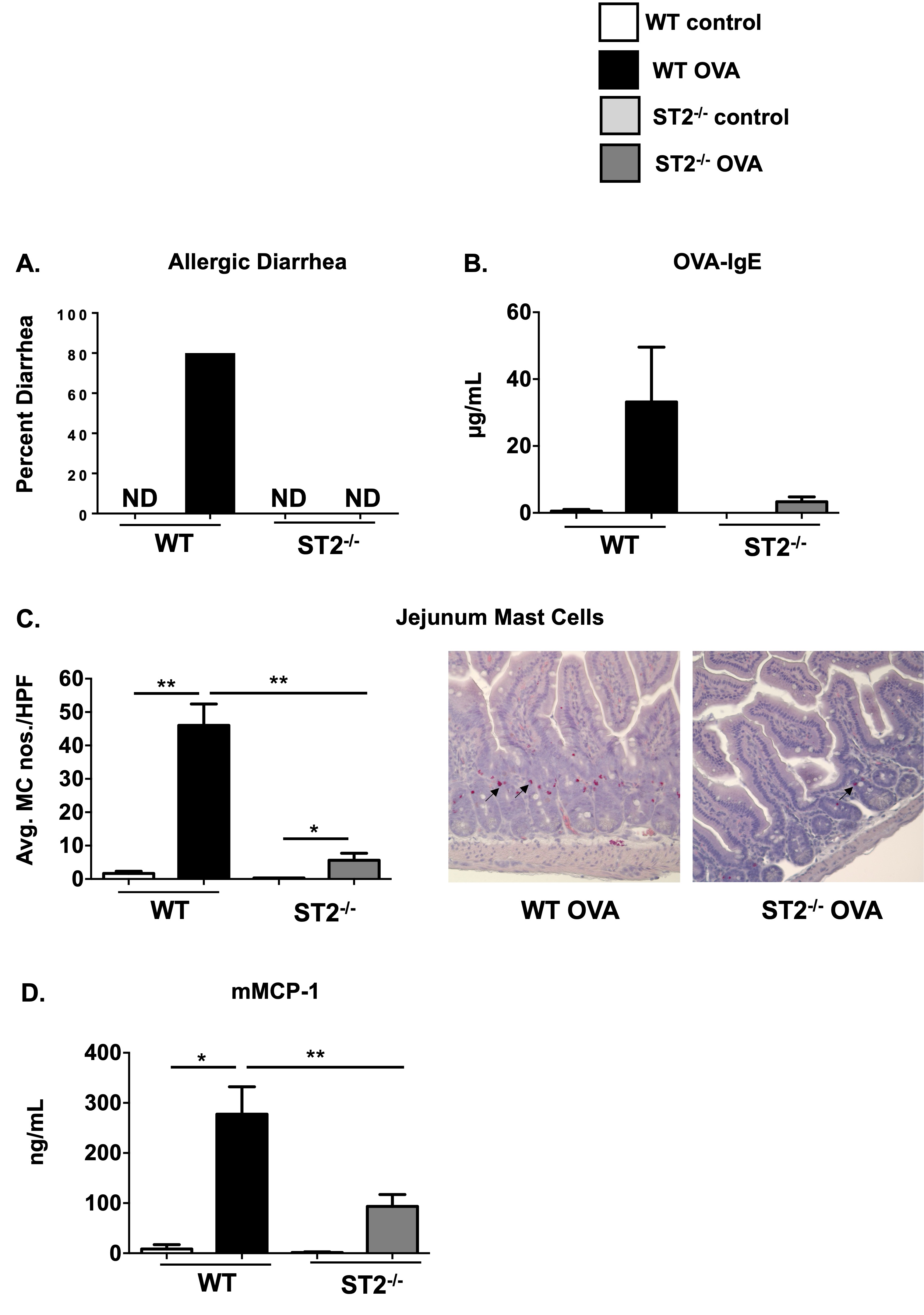

Supplement: Supplementary Figure 2 — MC responses and food allergy development are attenuated in ST2-/- mice. BALB/c and ST2-/- mice were sensitized and challenged with OVA as described in Methods. One hour after the 6th challenge, the following parameters were measured: (A) occurrence of diarrhea; (B) serum OVA-IgE levels; (C) CAE+ MCs in the jejunum (magnification: 40X; arrows depict MCs); (D) serum mMCP-1 levels. Data are representative of >3 experiments. n=5-7 mice/group. ND, not detected. *p<0.05; **p<0.01 (t-test). [file Image2.jpeg]

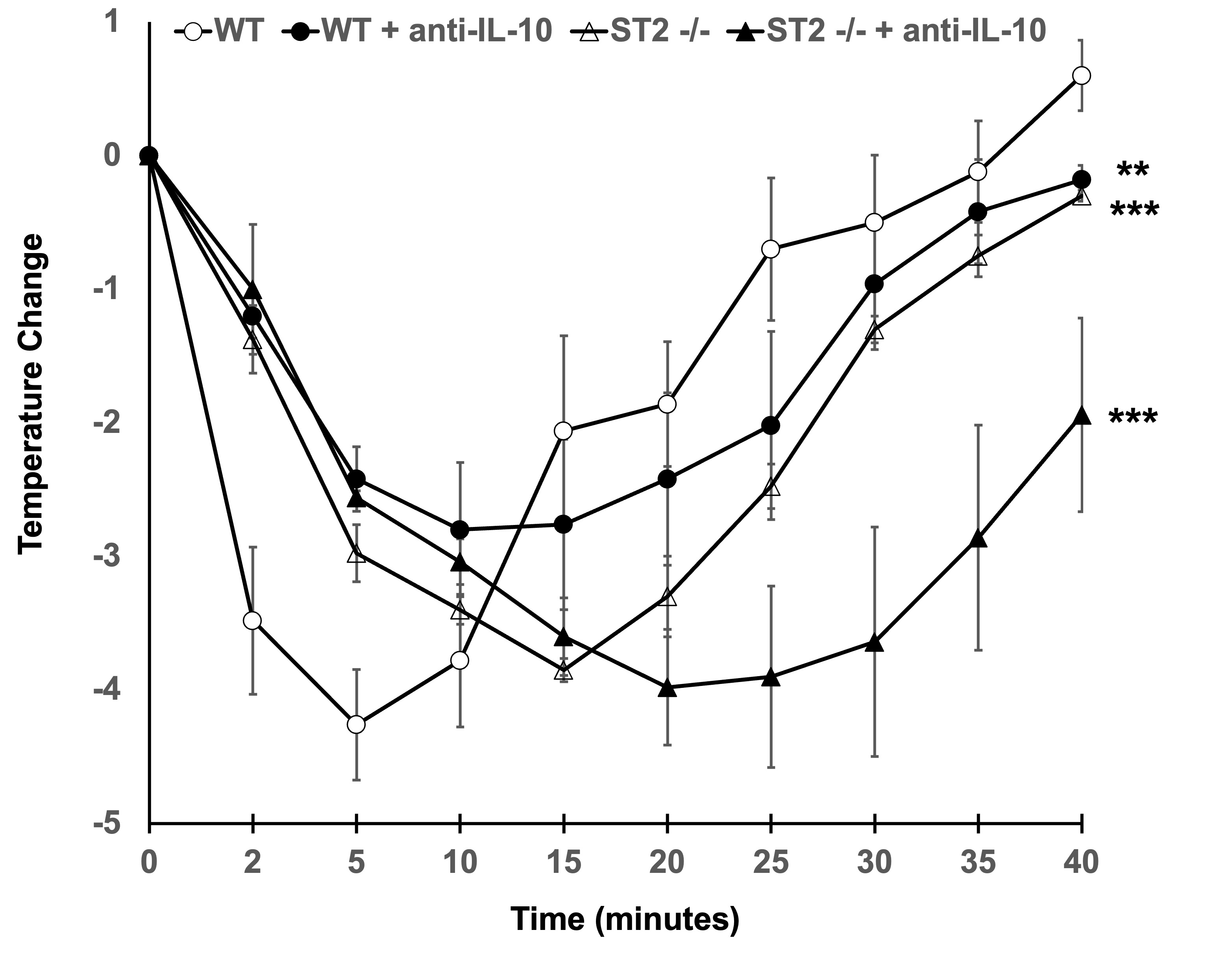

Supplement: Supplementary Figure 3 — Anti-IL-10 treatment attenuates IgE-mediated passive anaphylaxis in WT mice. Naïve BALB/c and ST2-/- mice were injected i.v. with 6 μg DNP-IgE. Some groups of mice were treated i.p. with 400 μg anti-IL-10. 24h later, all mice were injected i.v. with 75 μg DNP-BSA and changes in body temperature were measured. n=4-5 mice/group. **p<0.05 for the WT group compared with WT plus anti-IL-10-treated mice by 2-way ANOVA; ***p<0.05 for the WT group compared with ST2-/- mice and the ST2-/- group compared with anti-IL-10-treated ST2-/- mice by 2-way ANOVA. [file Image3.jpeg]
